# Identification of novel *JAK3* variants in a suspected SCID patient and two couples undergoing carrier screening

**DOI:** 10.3389/fgene.2026.1624523

**Published:** 2026-06-01

**Authors:** Yao Peng, Yi-Lin Sang, Wu Zhu, Ning Zhang, Yi Sun, Ge Lin, Guang-Xiu Lu, Yue-Qiu Tan, Juan Du, Fu-Yan Wang, Wen-Bin He

**Affiliations:** 1 Hunan Guangxiu Hospital Affiliated with Hunan Normal University, Hunan Normal University Health Science Center, Changsha, Hunan, China; 2 Department of Immunology, Xiangya School of Basic Medical Science, Central South University, Changsha, Hunan, China; 3 Institute of Reproductive and Stem Cell Engineering, NHC Key Laboratory of Human Stem Cell and Reproductive Engineering, Xiangya School of Basic Medical Sciences, Central South University, Changsha, Hunan, China; 4 National Engineering and Research Center of Human Stem Cells, Changsha, Hunan, China; 5 Clinical Research Center for Reproduction and Genetics in Hunan Province, Reproductive and Genetic Hospital of CITIC-Xiangya, Changsha, Hunan, China

**Keywords:** functional analyses, jak3, pathogenicity assessment, severe combined immunodeficiency, variants of uncertain significance

## Abstract

**Background:**

Severe combined immunodeficiency (SCID) is one of the most severe forms of primary immunodeficiency. *JAK3* gene is a critical determinant of SCID, as JAK3-STAT pathway regulates development, proliferation, activation, and differentiation of immune cells. This study aimed to identify the genetic cause of a family with a suspected SCID patient, and to perform carrier screening for two couples to assess the risk of conceiving offspring with birth defects.

**Methods:**

Whole-Exome Sequencing was performed on five individuals from the three families. A series of *in vitro* functional experiments, including Western blotting and luciferase assays, were conducted to assess the pathogenicity of the identified *JAK3* variants.

**Results:**

We identified seven *JAK3* variants, including five variants of uncertain significance (p.Arg402His, p.ILe688Phe, p.Leu129Phe, p.Met235Thr, p.Ala634Pro) and one pathogenic variant and one likely pathogenic variant (p.Gln1007Ter and p.Cys376Leufs*34). Among these, four variants (p.Gln1007Ter, p.Leu129Phe, p.Cys376Leufs*34 and p.Ala634Pro) were novel. *In vitro* functional experiments revealed that three of five variants of uncertain significance (VUSs) significantly reduced STAT5 phosphorylation and transcriptional activity, thereby reclassifying two variants (p.Arg402His and p.ILe688Phe) as likely pathogenic variants (LP) and one variant (p.Leu129Phe) as VUS with a Bayesian score of 3. In contrast, the remaining two variants (p.Ala634Pro and p.Met235Thr) did not affect JAK3 function, and were reclassified as VUS with a Bayesian score of 1 or 0.

**Conclusion:**

This study identified seven *JAK3* variants from three families, including four novel variants. Functional experiments revealed that two VUSs were reclassified as LP and one VUS were reclassified as VUS with a Bayesian score of 3. These findings highlight the importance of integrating genetic and functional analyses to enhance diagnostic accuracy, inform treatment strategies for patients, clarify of the risk for carrier-screening couples, improve genetic counseling, and guide reproductive interventions.

## 1 Introduction

Severe combined immunodeficiency (SCID) is a life-threatening form of primary immunodeficiency characterized by defective T-cell and NK-cell development and/or dysfunctional B-cell immaturity, resulting in severe impairments in both cellular and humoral immunity (Justiz-Vaillant et al., 2023; Aranda et al., 2024). The estimated incidence of SCID ranges from approximately 1/58,000 to 1/100,000 live births (Kwan et al., 2014; Aranda et al., 2024). Typically manifesting in infancy, SCID is clinically marked by recurrent infections, chronic diarrhea, failure to thrive, lymphopenia, and profound deficiencies in cellular and antibody-mediated immunity. Without intervention, such as hematopoietic stem cell transplantation, this condition is usually fatal within the first year of life (Dvorak et al., 2023). SCID is inherited in an X-linked recessive or autosomal recessive manner, with mutations in genes such as *IL2RG, RAG1, RAG2* and *JAK3* (Platt et al., 2021).

*JAK3* is a critical gene implicated in SCID. The encoded JAK3 protein interacts with the intracellular domain of the IL-2 receptor common gamma chain (IL2RG/γ_c_) (Liongue et al., 2024), facilitating the activation of signal transducers and activators of transcription (STAT) proteins (Klatzmann and Abbas, 2015; Luo et al., 2023). Cytokines signaling via the JAK3-STAT pathway exert pleiotropic effects on both innate and adaptive immunity, regulating development, proliferation, activation, and differentiation of T cells, B cells, and NK cells (Liang et al., 2012; Patidar et al., 2016; Morris et al., 2018; Leonard et al., 2019; Liongue et al., 2024). Biallelic mutations in the *JAK3* disrupt the JAK3-STAT pathway, causing SCID (Di Matteo et al., 2018; Wang et al., 2023; Sbruzzi et al., 2024). To date, a total of 151 mutations have been identified in patients and cataloged in the HGMD database; however, further functional evidence is required to confirm their pathogenicity and causal role in SCID. In clinical practice, genetic testing of suspected SCID patients or expanded carrier screening for monogenic disorders often identifies numerous of variants of uncertain significance (VUS) in the *JAK3*. Determining the pathogenicity of these VUS is essential for guiding patient treatment, assessing familial reproductive risks, and enabling prenatal diagnosis or preimplantation genetic testing for reproductive intervention.

In this study, we identified five *JAK3* gene VUSs, one likely pathogenic (LP) variant and one pathogenic (P) variant by high‐throughput sequencing in a family with a suspected SCID patient and in two couples undergoing carrier screening. Subsequently, we conducted a series of *in vitro* functional experiments that demonstrated whether these variants impaired the function of JAK3 protein. As a result, two VUSs were reclassified as LP. This study not only provides a theoretical basis for precision diagnosis, genetic counseling, and reproductive intervention for these families, but also highlights the critical role of functional analysis in reclassifying VUS.

## 2 Materials and methods

### 2.1 Patients

We recruited three unrelated Han Chinese families from the Reproductive and Genetic Hospital of CITIC-Xiangya, including one family with a suspected SCID patient and two couples seeking carrier screening for monogenic diseases. Patient from the family was initially diagnosed with SCID based on the following criteria: 1) age <2 years; 2) recurrent severe infections within the first few months of life that did not respond to a full course of antibiotics; 3) absence of growth retardation or disseminated BCG infection; and 4) absolute CD3^+^ T-lymphocyte counts <3 × 10^9^/L. This study was approved by the Ethics Committee of the Reproductive and Genetic Hospital of CITIC-Xiangya (LL-SC-2019-033). Because the patients from Family II and Family III were deceased, no biological specimens could be obtained. Written informed consent was obtained from all participants or the guardians.

### 2.2 High-Throughput Sequencing

Genomic DNA samples were extracted from the peripheral blood of subjects (patients and his parents in family I, the couples in families II and III) using the QIAamp DNA Blood Mini Kit (Qiagen, 51185). Whole exome sequencing was performed by Shenzhen BGI, and gene panel sequencing was performed by Guangzhou AmCare Genomics, following the protocol outlined in our previous study (He et al., 2018). In family I, candidate causative variants were identified based on the filtering strategy described in our earlier work (Tan et al., 2019), with a particular focus on genetic variants associated with severe combined immunodeficiency (SCID). In couples undergoing carrier screening, attention was given to variants in autosomal recessive genes shared by both spouses, as well as to variants in X-linked genes carried by the female partner. The pathogenicity of the identified variants was classified according to the American College of Medical Genetics and Genomics (ACMG) and the Association for Molecular Pathology (AMP) criteria, incorporating a range of evidence types (e.g., population data, computational predictions, functional studies, and segregation data) as detailed in the guidelines (Richards et al., 2015).

### 2.3 Plasmids

A polynucleotide fragment encompassing the entire coding region of human JAK3 (NM_000215.4) and IL2RG (NM_000206.3) was amplified by PCR using human peripheral blood cDNA as the template and ligated into the mammalian expression vector pCMV, leading to the production of Myc followed by JAK3 or IL2RG followed by 3XFLAG, as pCMV-Myc-*JAK3*-WT and pCMV-IL2RG-3×FLAG-WT plasmids. *JAK3* variant plasmids were generated using the Mut Express® II Fast Mutagenesis Kit (Vazyme, C214), with the pCMV-Myc-*JAK3*-WT plasmid as the template, following the manufacturer’s instructions. All wild-type and variant plasmids were verified by Sanger sequencing.

### 2.4 Cell culture and transfection

HEK293 cells were cultured in SMM 293-TII complete medium (Sino Biological Inc., M293TII) containing 10% fetal bovine serum (Gibco, A3160901) and 1% streptomycin-penicillin solution in T25 cell culture flasks at 37 °C in a 5% CO_2_ incubator. When cells grew to 70%–80% confluence, they were transiently transfected with pCMV-Myc-*JAK3*-WT or variant plasmids using Neofect DNA Transfection Reagent (Neofect, TF20121201) according to the manufacturer’s instructions.

### 2.5 Western blotting

To confirm the expression of pCMV-Myc-*JAK3*-WT and variant plasmids, protein blotting was performed. HEK293 cells were transiently transfected with pCMV-Myc-*JAK3*-WT or variant plasmids and harvested 48 h post-transfection. Proteins extracted from the transfected cells were blotted onto polyvinylidene difluoride (PVDF) membranes (Merck Millipore, 0000187588) and incubated with Myc-tag antibody (1:1000, Abways, AB0001) overnight at 4 °C. The following day, the membranes were incubated with the secondary antibody (1:10000, Abways). Finally, the proteins were visualized using the Omni-ECL™ Ultrasensitive Chemiluminescence Detection Kit (Epizyme Biotech, SQ201L).

### 2.6 STAT5 phosphorylation and luciferase assays

STAT5 phosphorylation was assessed in HEK293 cells co-transfected with Myc-tagged *JAK3* (WT or variant) and Flag-tagged IL2RG plasmids. Following stimulation with Recombinant Human IL-2 (1000 U/mL, peprotech, 200-02) for 30 min, IL-2-stimulated cells were harvested and lysed with RIPA buffer containing PMSF (Biosharp, BL507A) and a protein phosphatase inhibitor (Solarbio, P1260) (Solarbio, R0020) lysis. The lysates were centrifuged at 12,000 g for 10 min at 4 °C and the supernatant was used for immunoblotting analysis. The PVDF membranes were incubated overnight at 4 °C with primary antibodies against Myc-tag (1:1000, Abways, AB0001), Flag-tag (1:1000, Cell Signaling Technology, 8146), Stat5 (1:1000, Cell Signaling Technology, 94205), Phospho-Stat5 (1:1000, Cell Signaling Technology, 4322) and GAPDH (1:1000, Affinity, T0004).

For the STAT5 reporter gene assay, HEK293 cells were plated in 24-well plates and transiently co-transfected with *JAK3* (WT or variant) plasmid, flag-tagged IL2RG vector and pGL4.52 (Luc2P/STAT5RE/Hygro) reporter gene plasmid (Promega, E465A). Thirty-six hours after transfection, IL-2 (1000 U/mL) was added to the culture and incubated for 40 min. Cells were then lysed, and luciferase activity was measured using One-Glo luciferase detection reagent (Promega) according to the manufacturer’s recommendations.

The pathogenicity of all variants was reclassified in accordance with the ACMG/AMP criteria and the Bayesian framework (Richards et al., 2015; Ellard et al., 2020).

### 2.7 Statistical analysis

All experiments were independently repeated at least three times (n ≥ 3), and each group was compared with the JAK3-WT group (*p < 0.05, **p < 0.01, ***p < 0.001, **** indicates *p* < 0.0001, ns indicates not significant). Statistical analyses were performed using Student’s t-test and one-way analysis of variance (ANOVA) using SPSS software (version 20.0, Chicago, IL, United States of America).

## 3 Results

### 3.1 Case presentation

In Family I, the child experienced recurrent lung infections, accompanied by eczema, thrush, skin abscess and delayed growth and development. Lymphocyte subtypes analysis showed reduced CD3^+^T cells, CD3^+^CD4^+^T cells and CD3^+^CD8^+^T cells, while the ratio of CD4^+^/CD8^+^T cells and the number of CD19^+^B cells were elevated. Despite repeated treatments, the child passed away at the age of 11 months. The couple requested a genetic test for the patient.

In Family II, the couple had a history of two pregnancies. The first pregnancy resulted in intrauterine fetal death at 36 weeks due to placental abruption. In the second pregnancy, labor was induced at 26 weeks of gestation due to abnormal cardiovascular development, including ventricular septal defect and aortic straddle. Since samples from the dead infants were unavailable, the couple requested carrier screening for monogenic genetic diseases.

In Family III, the child presented with widespread eczema at the age of 1 month, which showed minimal improvement despite the application of topical treatment. At 2 months of age, he exhibited decreased breastfeeding, diarrhea and severe pulmonary wheezing. The child unfortunately passed away at approximately 4 months of age. Since samples from the child were unavailable, the parents requested carrier screening for monogenic genetic diseases.

### 3.2 Genetic analysis

**FIGURE 1:**
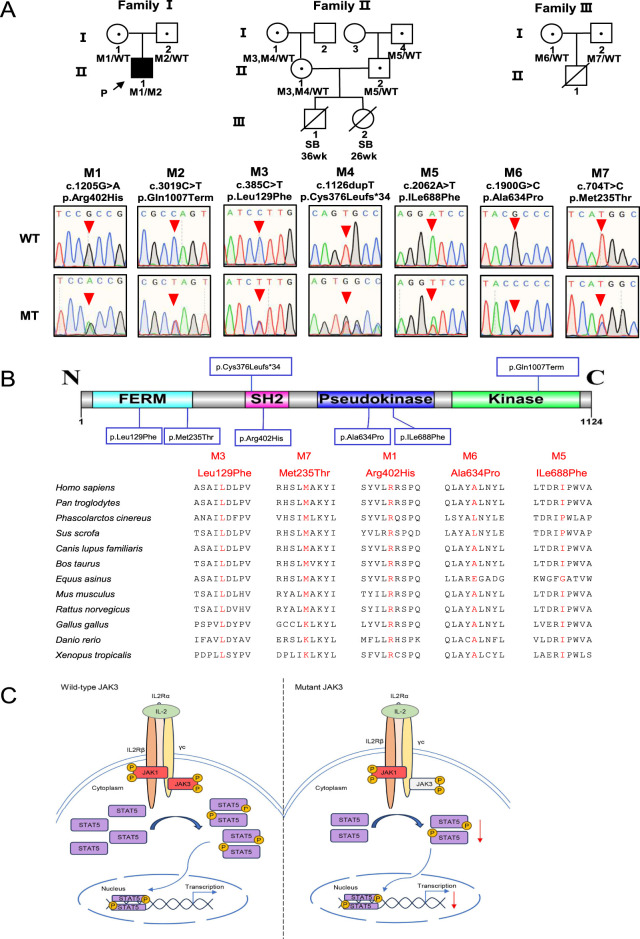
Identification of *JAK3* variants in a suspected SCID patient and two couples undergoing carrier screening. **(A)** Pedigree and genetic analysis of *JAK3* in a suspected SCID patient and two couples undergoing carrier screening. M3 and M4 are in cis. The black arrow symbol represents proband. Sanger sequencing chromatograms of the *JAK3* variants (M1-M7) are shown at the bottom. The red arrowhead represents positions of *JAK3* variants. **(B)** Locations and conservation analysis of the identified variants in *JAK3*. The positions of all variants are indicated in the protein structure of JAK3. The comparison of species indicates the conservation of missense mutated amino acids. **(C)** JAK3-STAT5 pathways. After interleukin-2 (IL-2) binds to its receptor, this signal activates the associated Janus kinases JAK1 and JAK3, which trans-phosphorylate each other and phosphorylate the receptor cytoplasmic tails to create docking sites. The activated JAKs subsequently activates STAT5. Tyrosine-phosphorylated STAT5 (p-STAT5) dimerizes, translocate to the nucleus, bind cognate DNA elements, and regulate target gene transcription. The mutant JAK3 disrupts the interaction between the IL‐2Rγ_c_ (IL2RG) and JAK3 protein, decreases STAT5 phosphorylation and consequently reduces downstream gene expression.

We performed high‐throughput sequencing on the proband in family I and the parents from family II and III. In family I, the patient exhibited compound heterozygous *JAK3* variant, c.3019C>T(p.Gln1007Ter) and c.1205G>A(p.Arg402His), which were inherited from his father and mother, respectively. In family II, the man carried the heterozygous variant c.2062A>T(p.ILe688Phe), while the woman carried two heterozygous variants c.385C>T(p.Leu129Phe) and c.1126dupT(p.Cys376Leufs*34), which were in cis and both inherited from her mother. In family III, the male and female carried the heterozygous variants c.704T>C(p.Met235Thr) and c.1900G>C(p.Ala634Pro), respectively (Figure 1A). Among these, four variants (p.Gln1007Ter, p.Leu129Phe, p.Cys376Leufs*34 and p.Ala634Pro) were novel. The three missense variants (p.Arg402His, p.ILe688Phe and p.Leu129Phe) were highly conserved across species, while the other two missense variants were not conserved (Figure 1B). In addition, prediction tools, including PolyPhen-2, CADD, MutationTaster, REVEL and AlphaMissense, predicted that most variants were pathogenic, except for two variants (p.Leu129Phe and p.Met235Thr), which were predicted as benign (Table 1). According to the American Society of Medical Genetics and Genomics (ACMG) standard guide for sequence variation interpretation, p.Gln1007Ter and p.Cys376Leufs*34 variants were classified as P variants, and the remaining five *JAK3* missense variants were classified as VUS.

**TABLE 1:** Detailed description of the variants in *JAK3* identified in three families.

Family	Gene	Location [Hg19]	RefSeq ID	Exon	Variant	Variant type	1000 Genomes	gnomAD	gnomAD-EAS	PolyPhen-2^a^	CADD^b^	Mutation Taster^c^	REVEL^d^	AlphaMissense^e^
I	*JAK3*	chr19:17951088	NM_000215.3	9	c.1205G>A(p.Arg402His)	Missense	NA	3.987e-6	0	PD	29.3	Deleterious	0.5	0.4663
		chr19:17941389	NM_000215.3	22	c.3019C>T(p.Gln1007Ter)	Nonsense	NA	NA	NA	-	40	Deleterious	-	-
II	*JAK3*	chr19:17954224	NM_000215.3	4	c.385C>T(p.Leu129Phe)	Missense	NA	NA	NA	Benign	12.94	Benign	0.154	0.1712
		chr19:17952214	NM_000215.3	8	c.1126dupT(p.Cys376Leufs*34)	Frameshift	NA	NA	NA	-	34	Deleterious	-	-
		chr19:17945798	NM_000215.3	16	c.2062A>T(p.ILe688Phe)	Missense	6.039e-4	2.265e-4	3.161e-3	PD	27	Deleterious	0.774	0.83
III	*JAK3*	chr19:17946747	NM_000215.3	14	c.1900G>C(p.Ala634Pro)	Missense	NA	NA	NA	PD	31	Deleterious	0.779	0.964
		chr19:17953282	NM_000215.3	6	c.704T>C(p.Met235Thr)	Missense	NA	NA	NA	Benign	22.9	Benign	0.236	0.4814

a PloyPhen-2, score: ranges from 0.000 to 1.000, and 0.000 is benign and 1.000 is damaging. PD: Probably_damaging.

b CADD, score: amino acid substitution is predicted damaging if the score is > 15.

c MutationTaster: the probability value refers to the prediction, i.e., a value close to 1 indicates a high “security” of the prediction.

d REVEL, score: ≥0.644 is considered pathogenic supporting, 0.291–0.643 is neutral, and ≤0.290 is benign supporting.

e AlphaMissense score: ≥0.564 is considered likely pathogenic, 0.340–0.565 is likely neutral, ≤0.340 is likely benign.

NA: not available.

### 3.3 *JAK3* variants resulted in reduced IL-2-induced STAT5 phosphorylation and transcriptional activity

**FIGURE 2:**
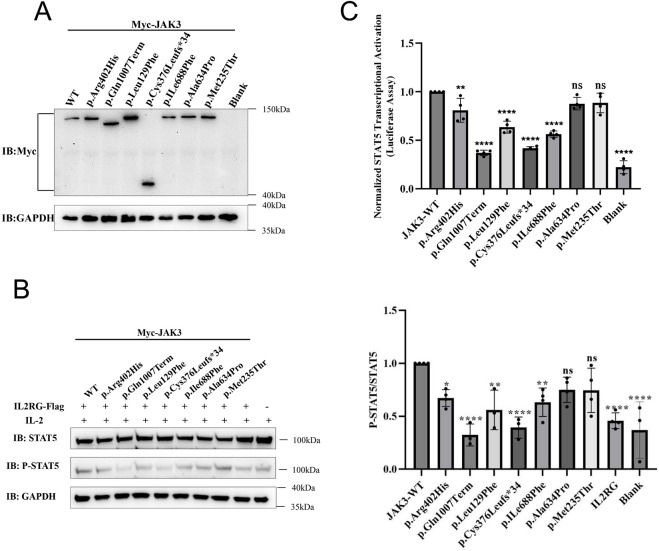
Function analysis of these identified *JAK3* variants *in vitro*. **(A)** Western blot was used to detect the protein expression of wild and *JAK3* variant plasmids. **(B)** Cells were stimulated with 1000 U/mL IL-2 stimulation. Western blot analysis of p-STAT5 and total STAT5 protein expression was shown in left panel. The right panel presented a statistical graph. **(C)** Luciferase reporter assay was used to measure STAT5 transcriptional activity. All experiments were independently repeated at least three times (n ≥ 3), and each group was compared with the JAK3-WT group (*p < 0.05, **p < 0.01, ***p < 0.001, ****p < 0.0001, ns for not significant).

The binding of JAK3 to γ_c_ is necessary for STAT5 phosphorylation (Arcas-García et al., 2020). The mutant JAK3 disrupts the interaction between the IL2RG and JAK3 protein, decreases STAT5 phosphorylation and consequently reduces downstream gene expression (Figure 1C). To investigate whether *JAK3* variants impair the STAT5 signal transduction, the *JAK3* (wild-type and variant) plasmids were constructed and were transfected into HEK293 cells, confirming successful expression of *JAK3* (Figure 2A). Subsequently, we examined IL-2-induced STAT5 phosphorylation by Western blot analysis. Under stimulation with 1000 U/mL IL-2, STAT5 phosphorylation in cells transfected with five variants plasmids (p.Arg402His, p.Gln1007Ter, p.Leu129Phe, p.Cys376Leufs*34 and p.Ile688Phe) were significantly decreased compared to cells transfected with wild-type *JAK3* plasmid in Western blot analysis. Among these, the p.Gln1007Ter and p.Cys376Leufs*34 JAK3 resulted in the most pronounced decrease in STAT5 phosphorylation, while no significant difference in STAT5 phosphorylation was observed in cells transfected with p.Ala634Pro and p.Met235Thr compared to wild-type *JAK3* plasmid (Figure 2B).

In addition, luciferase reporter assays revealed a significant reduction in STAT5 transcriptional activity in cells expressing the five variant plasmids, whereas cells transfected with the other two variant plasmids (p.Ala634Pro and p.Met235Thr) maintained the STAT5 activity levels comparable to wild-type *JAK3* (Figure 2C). These findings suggested that the five *JAK3* variants (p.Arg402His, p.Gln1007Ter, p.Leu129Phe, p.Cys376Leufs*34 and p.ILe688Phe) impaired JAK3 function, whereas p.Ala634Pro and p.Met235Thr likely did not disrupt JAK3 function.

### 3.4 Reclassification of *JAK3* variants pathogenicity

Initially, according to the ACMG guidelines, we identified five JAK3 VUSs, one LP variant and one P variant on a suspected SCID family and two carrier-screening families. After a series of *in vitro* functional analyses, a significant reduction in STAT5 phosphorylation and transcriptional activity was observed in cells expressing the five mutant JAK3 proteins, comprising one P variant, one LP variant and three VUSs, whereas the other two JAK3 VUSs (p.Ala634Pro and p.Met235Thr) did not impair JAK3 function. Finally, among the five VUSs, two variants (p.Arg402His and p.ILe688Phe) were reclassified as LP, while one variant (p.Leu129Phe) was reclassified as VUS with a Bayesian score of 3. The other two VUSs (p.Ala634Pro and p.Met235Thr) were reclassified as VUS with Bayesian scores of 1 or 0. The detailed data is presented in Table 2.

**TABLE 2:** Reclassification of the seven JAK3 variants.

Family	Gene	Variant	Original classification/Scores	Reclassification/Scores
I	*JAK3*	c.1205G>A(p.Arg402His)	VUS(PM2_Supporting + PM3+PP4)/4	LP(PM2_Supporting + PM3+PP4+PS3_Moderate)/6
		c.3019C>T(p.Gln1007Ter)	P(PM2_Supporting + PVS1+PP4)/10	P(PM2_Supporting + PVS1+PP4)/10
II	*JAK3*	c.385C>T(p.Leu129Phe)	VUS(PM2_Supporting)/1	VUS(PM2_Supporting + PS3_Moderate)/3
		c.1126dupT(p.Cys376Leufs*34)	LP(PM2_Supporting + PVS1)/9	LP(PM2_Supporting + PVS1)/9
		c.2062A>T(p.ILe688Phe)	VUS(PM1+PM2_Supporting + PP3)/4	LP(PM1+PM2_Supporting + PP3+PS3_Moderate)/6
III	*JAK3*	c.1900G>C(p.Ala634Pro)	VUS(PM2_Supporting + PP3)/2	VUS(PM2_Supporting + PP3+BS3_Supporting)/1
		c.704T>C(p.Met235Thr)	VUS(PM2_Supporting)/1	VUS(PM2_Supporting + BS3_Supporting)/0

The type and strength of the of evidence in the American College of Medical Genetics and Genomics (ACMG) standards and guidelines for the interpretation of variants. This study employed the ACMG/AMP-compatible point system. Within this system, the final scores are based on the cumulative scores of each independent evidence in the ACMG, guidelines. The system is constructed based on Bayes’ rule and the ACMG/AMP, variant classification guidelines. Detailed scores: PVS1 (Pathogenic Very Strong, +8), PS (Pathogenic Strong, +4), PM (Pathogenic Moderate, +2), PP (Pathogenic Supporting, +1), BS (Benign Strong, −4); P ≥ 10, 6≤LP ≤ 9, 0≤VUS≤5, −6≤LB ≤ −1, B < −6.

Abbreviations: VUS, variant of uncertain significance; LP, likely pathogenic; P, pathogenic.

## 4 Discussion

*JAK3* is localized to 19p13.11 and encodes a tyrosine kinase protein (JAK3) predominantly expressed in hematopoietic cells. It transduces signals downstream of IL2RG, which is shared by multiple cytokine receptors, including those for IL-2, IL-4, IL-7, IL-9, IL-15, and IL-21 (Lin and Leonard, 2018; Liongue et al., 2024). Upon cytokine binding, receptor conformational changes and aggregation of associated JAK proteins, which in turn drives tyrosine transphosphorylation, activating JAK kinase domains and signaling pathways such as STAT5 and STAT6 (Wu and Sun, 2012). When JAK3 protein function is impaired, it results in disrupting IL2RG receptor-mediated signaling, impairing STAT5 and STAT6 activation, and leading to defective lymphocyte development, and SCID (Bogaert et al., 2016; Pan et al., 2022; Sbruzzi et al., 2024). Approximately 7%–14% of SCID cases are caused by pathogenic variants in the *JAK3* gene (Aluri et al., 2019; Pan et al., 2022). In this study, we identified five *JAK3* VUSs, one LP variant and one P variant by high-throughput sequencing on a suspected SCID family and two carrier-screening families, including four novel variants (p.Gln1007Ter, p.Leu129Phe, p.Cys376Leufs*34 and p.Ala634Pro). Among the families studied, patients from Families II and III were deceased, leading to a lack of available biological specimens. A weak correlation was observed between genotype and phenotype. The purpose of two-family studies is to facilitate the classification of functional variants for carrier risk assessment. According to the ACMG guidelines and a series of *in vitro* functional analyses, we finally identified three VUS variants (p.Leu129Phe, p.Ala634Pro and p.Met235Thr), three LP variants (p.Arg402His, p.Ile688Phe and p.Cys376Leufs*34), and one P variant (p.Gln1007Ter) (Table 2). The results clarify the genetic etiology of family I and provide a theoretical basis for fertility counselling in these three families.

The signal transducer and activator of transcription STAT5 plays a crucial role in immune regulation by mediating three sequential tyrosine phosphorylation triggered by cytokine stimulation through the JAK-STAT pathway, a process essential for lymphocyte development and differentiation (Morris et al., 2018; Maurer et al., 2019). Previous studies have shown that although IL-2 and IL-4 are both γ_c_ cytokines, they predominantly activate different downstream STAT pathways: IL-2 predominantly activates STAT5A/STAT5B, whereas IL-4 primarily activates STAT6 (Leonard et al., 2019). In cases of SCID due to *JAK3* variants, JAK3 mutant proteins were observed to significantly reduced IL-2-induced STAT5 phosphorylation (Leonard et al., 2019). In this study, we used IL-2 to stimulate the cells and then detected the phosphorylation level of STAT5 in the cells to evaluate the function of JAK3. Western blot analysis and luciferase reporter gene analyses revealed that cells transfected with five *JAK3* variant plasmids (p.Arg402His, p.Gln1007Ter, p.Leu129Phe, p.Cys376Leufs*34, and p.Ile688Phe) exhibited reduced STAT5 phosphorylation and reduced transcriptional activity compared to wild-type JAK3-transfected cells, suggesting these variants impaired the function of JAK3 protein. In contrast, cells transfected with the remaining two variants showed no significant difference in IL-2-induced STAT5 phosphorylation or transcriptional activity compared to wild-type controls. These findings underscore the importance of assessing IL-2-induced STAT5 phosphorylation and transcriptional activity as a robust method for evaluating the pathogenicity of *JAK3* variants.

The JAK3 protein consists of four functional domains, including the FERM domain, the Src-homology 2 (SH2) domain, the pseudokinase domain (JH2), and the kinase domain (JH1) (Hu et al., 2021). The SH2 and FERM domains are mainly responsible for mediating binding to the cytokine-receptor membrane-proximal box1/2 regions and stabilizing the receptor-JAK complex (Ferrao and Lupardus, 2017). The kinase domain is responsible for tyrosine phosphorylation and activates downstream STAT proteins (Hu et al., 2021). The pseudokinase domain lacks kinase activity, but it plays a role in mediating the interaction between JAK and STAT and regulating the activity of the kinase domain. In this study, two variants (p.Leu129Phe and p.Arg402His) are respectively located in FERM and SH2 domains, which affected the interaction between JAK3 FERM-SH2 and IL2RG, and eventually led to a decrease in STAT5 phosphorylation. The variant, p.ILe688Phe, might compromise JAK3 catalytic function and reduce STAT5 tyrosine phosphorylation. However, the p.Ala634Pro variant in another pseudokinase domain, even though predicted to be pathogenic, did not lead to STAT5 phosphorylation. This variant may be located in the non-critical region of the pseudokinase domain, where the substitution does not disrupt core function.

There are two limitations in this study. Firstly, a weak correlation was observed between genotype and phenotype in Families II and III, due to the patients were deceased. The purpose of the two-family studies is to facilitate the classification of functional variants for carrier risk assessment. Secondly, this study primarily investigated JAK3 function in HEK293 cells. Because HEK293 is a non-lymphoid line whose intracellular signaling milieu differs from that of native lymphocytes, our findings may not fully reflect the physiological role of JAK3 in T cell development, activation, and function. Future studies should validate these observations in primary CD4^+^ T cells.

In conclusion, this study identified seven *JAK3* variants in a family with SCID and two carrier-screening couples using high-throughput sequencing, four of which were novel. Initially, five variants were classified as VUSs. Functional analyses revealed that three of five VUSs reduced STAT5 phosphorylation and transcriptional activity-key processes for immune function-leading to their reclassification as LP or as VUS with a Bayesian score of 3. This work underscores the importance of integrating genetic and functional analyses to improve precision in diagnosis, inform treatment strategies for patients, clarify of the risk for carrier-screening couples, improve genetic counseling, and guide reproductive interventions.

## Data Availability

The original contributions presented in the study are included in the article. Further inquiries can be directed to the corresponding authors.
